# Endoplasmic reticulum stress cooperates with Toll-like receptor ligation in driving activation of rheumatoid arthritis fibroblast-like synoviocytes

**DOI:** 10.1186/s13075-017-1386-x

**Published:** 2017-09-18

**Authors:** Pawel A. Kabala, Chiara Angiolilli, Nataliya Yeremenko, Aleksander M. Grabiec, Barbara Giovannone, Desiree Pots, Timothy R. Radstake, Dominique Baeten, Kris A. Reedquist

**Affiliations:** 1 Department of Rheumatology and Clinical Immunology, University Medical Center Utrecht, Utrecht, The Netherlands; 2 Department of Clinical Immunology and Rheumatology, Academic Medical Centre/University of Amsterdam, Amsterdam, The Netherlands; 3 Amsterdam Rheumatology and Immunology Center, Amsterdam, The Netherlands; 4 Department of Experimental Immunology, Academic Medical Centre/University of Amsterdam, Amsterdam, The Netherlands; 5 Laboratory of Translational Immunology, University Medical Center Utrecht, Utrecht, The Netherlands; 6 Department of Microbiology, Faculty of Biochemistry, Biophysics and Biotechnology, Jagiellonian University, Krakow, Poland; 7 Division of Internal Medicine and Dermatology, Department of Dermatology/Allergology, University Medical Center Utrecht, Utrecht, The Netherlands

**Keywords:** ER stress, Rheumatoid arthritis, Fibroblast-like synoviocytes, Dermal fibroblasts, Macrophages, RNA stability, Inflammation

## Abstract

**Background:**

Endoplasmic reticulum (ER) stress has proinflammatory properties, and transgenic animal studies of rheumatoid arthritis (RA) indicate its relevance in the process of joint destruction. Because currently available studies are focused primarily on myeloid cells, we assessed how ER stress might affect the inflammatory responses of stromal cells in RA.

**Methods:**

ER stress was induced in RA fibroblast-like synoviocytes (FLS), dermal fibroblasts, and macrophages with thapsigargin or tunicamycin alone or in combination with Toll-like receptor (TLR) ligands, and gene expression and messenger RNA (mRNA) stability was measured by quantitative polymerase chain reaction. Cellular viability was measured using cell death enzyme-linked immunosorbent assays and 3-(4,5-dimethylthiazol-2-yl)-2,5-diphenyltetrazolium bromide assays, and signaling pathway activation was analyzed by immunoblotting.

**Results:**

No cytotoxicity was observed in FLS exposed to thapsigargin, despite significant induction of ER stress markers. Screening of 84 proinflammatory genes revealed minor changes in their expression (fold change 90th percentile range 2.8–8.3) by thapsigargin alone, but the vast majority were hyperinduced during combined stimulation with thapsigargin and TLR ligands (35% greater than fivefold vs lipopolysaccharide alone). The synergistic response could not be explained by quantitative effects on nuclear factor-κB and mitogen-activated protein kinase pathways alone, but it was dependent on increased mRNA stability. mRNA stabilization was similarly enhanced by ER stress in dermal fibroblasts but not in macrophages, correlating with minimal cooperative effects on gene induction in macrophages.

**Conclusions:**

RA FLS are resistant to apoptosis induced by ER stress, but ER stress potentiates their activation by multiple TLR ligands. Interfering with downstream signaling pathway components of ER stress may be of therapeutic potential in the treatment of RA.

**Electronic supplementary material:**

The online version of this article (doi:10.1186/s13075-017-1386-x) contains supplementary material, which is available to authorized users.

## Background

Genetic studies have shed light on our understanding of the causes of autoimmune diseases by identifying shared and unique risk loci among these diseases. However, in rheumatoid arthritis (RA), only a fraction of disease susceptibility can be explained by genetic variation [1], and the temporal link between the break of self-tolerance and development of clinical disease remains elusive because circulating autoantibodies are detectable long before the onset of arthritis [2]. In RA, stromal cells in the joint, fibroblast-like synoviocytes (FLS), exhibit an imprinted and epigenetically maintained aggressive phenotype, predisposing them to participate in an inflammatory positive feedback loop in response to the cues from the synovial environment [3]. Identifying local tissue conditions able to initiate and perpetuate the ensuing inflammatory cycle is therefore of critical importance to understanding and intervening in the disease process.

Endoplasmic reticulum (ER) stress is a common cellular response to many of the conditions RA FLS encounter in the inflamed synovium [4], and it occurs when the amount of newly synthesized proteins in the ER exceeds the organelle’s capacity to ensure their proper folding. The resulting accumulation of misfolded proteins in the ER triggers a set of signals collectively referred to as the *unfolded protein response* (UPR), aimed at relieving the burden by slowing down the global translation rate while increasing production of a selected set of proteins, particularly ER chaperones [5]. The UPR depends upon the triggering of inositol-requiring enzyme 1α (IRE1α), protein kinase R-like endoplasmic reticulum kinase (PERK), and activating transcription factor 6, sensors embedded in the ER membrane, by unfolded protein aggregates in the lumen. In response, IRE1α homodimerizes and causes the unconventional splicing of X-box binding protein 1 (*XBP1*) messenger RNA (mRNA). This causes a frameshift mutation in *XBP1*, making it a powerful transcription factor instrumental in restoring homeostasis. Additional transcription factors are activated by the two other sensors [6].

Although primarily a safeguard for protein folding homeostasis, ER stress is tightly associated with immunological processes via crosstalk occurring between the UPR and inflammatory signaling pathways. For example, the decrease in translation rate caused by PERK activity limits expression of proteins with a short half-life, such as nuclear factor of kappa light polypeptide gene enhancer in B-cells inhibitor, alpha (IκBα), resulting in enhanced activation of the nuclear factor (NF)-κB pathway [7]. Autophosphorylated IRE1α interacts with the tumor necrosis factor receptor-associated factor 2 adaptor molecule, facilitating activation of NF-κB and mitogen-activated protein (MAP) kinase pathways [8]. Transcription factors involved in ER stress can directly drive expression of inflammatory gene products such as interleukin (IL)-6 and tumor necrosis factor (TNF) [9], and elements of the UPR are necessary for maturation of several immune cell populations [10, 11]. Consequently, ER stress has been linked to a number of human disease conditions, including autoimmunity, where it has been postulated to drive inflammatory activation, act as the source of or as an adjuvant for autoantigens, or contribute to pathology by modulating apoptotic pathways [12, 13].

Despite this, understanding of the relevance of ER stress to pathology in RA is largely incomplete. Analysis of publicly available datasets of microarrays performed on synovial tissue has identified genes related to ER stress and protein processing in the ER as those most significantly differentiating between RA and osteoarthritis (OA) synovia, whereas no such difference was observed between OA and normal synovia [14]. Prominent staining for ER stress markers was observed throughout RA synovial tissue, particularly in the lining layer, indicating that these differences were unlikely to reflect changes in numbers of minor cell populations. A similar enhancement of ER stress and ER stress signaling to the nucleus in synovial fluid macrophages has been observed, and the ER chaperone binding immunoglobulin protein (BiP) is an important regulator of synovial angiogenesis, synoviocyte proliferation and survival, and disease severity in animal models of RA [14]. In experimental arthritis, strong expression of ER stress markers is observed during disease development in both synovial macrophages and fibroblasts [15]. Whereas myeloid-specific targeting of UPR pathways resulted in decreased cytokine expression and ameliorated disease in K/BxN serum-induced arthritis [16], studies involving RA FLS focus predominantly on changes in cellular viability and their potential consequences for synovial hyperplasia [17]. Unlike other cell types, RA FLS are resistant to apoptosis induced by ER stress, likely due to enhanced rates of autophagy and proteasomal activity [18, 19]. However, little is known about how ER stress changes the potential of FLS to directly modulate synovial inflammation, and recent studies have indicated that splicing of *XBP1* may be associated with the activation of RA FLS by Toll-like receptor (TLR) signaling, IL-1β, and TNF [20]. The aim of this study was therefore to examine if ER stress could regulate inflammatory gene expression in RA FLS.

## Methods

### Patients and cells

FLS were derived from synovial biopsies obtained by needle arthroscopy from patients fulfilling the 2010 American College of Rheumatology/European League Against Rheumatism classification criteria for RA [21, 22] and isolated as previously described [23]. Healthy skin biopsies were obtained as resected material after cosmetic surgery, and dermal fibroblasts (DF) were isolated using the Whole Skin Dissociation Kit (Miltenyi Biotec, Leiden, The Netherlands) following the manufacturer’s instructions. FLS and DF were cultured in DMEM (Gibco/Thermo Fisher Scientific, Waltham, MA, USA) containing 10% FBS (Invitrogen/Thermo Fisher Scientific) and used for experiments between passages 5 and 10. Prior to stimulations, cells were incubated in medium containing 1% FBS overnight.

Monocytes were isolated from healthy donor buffy coats (Sanquin, Amsterdam, The Netherlands) using Lymphoprep (AXIS-SHIELD; Alere Technologies, Oslo, Norway) density gradient centrifugation followed by standard isotonic Percoll gradient centrifugation (GE Healthcare, Eindhoven, The Netherlands). Monocytes were plated in Iscove’s modified Dulbecco’s medium (IMDM; Invitrogen/Thermo Fisher Scientific), supplemented with 1% FBS, for 30 minutes at 37 °C, followed by the removal of nonadherent cells. Monocytes were differentiated into macrophages by 7 days of culture in IMDM containing 10% FBS, 100 μg/ml gentamicin, and 800 U/ml granulocyte-macrophage colony-stimulating factor (Tebu-Bio, Heerhugowaard, The Netherlands).

### Cell stimulation

*Escherichia coli* 0111:B4 lipopolysaccharide (LPS) was ordered from Sigma-Aldrich (Zwijndrecht, The Netherlands) and used at 1 μg/ml. ER stress was induced by tunicamycin from *Streptomyces* sp. (10 μg/ml; Sigma-Aldrich) or thapsigargin at varying concentrations (Calbiochem/Merck, Amsterdam-Zuidoost, The Netherlands). Other stimulants used included IL-1β (1 ng/ml; R&D Systems, Minneapolis, MN, USA), polyinosinic:polycytidylic acid (pI:C; TLR3 agonist, 25 μg/ml; InvivoGen, San Diego, CA, USA), Pam3CSK4 (TLR1/2 agonist, 5 μg/ml; InvivoGen), flagellin (TLR5 agonist, 200 ng/ml; InvivoGen), SB202190 (p38 inhibitor,10 μM; Tocris Bioscience, Bristol, UK), U0216 (extracellular signal-regulated kinase [ERK] inhibitor, 10 μM; Tocris Bioscience), and c-Jun N-terminal (JNK) inhibitor IX (20 μM; Calbiochem/Merck).

### Gene expression measurement

Total RNA was isolated using an RNeasy Micro Kit (QIAGEN, Venlo, The Netherlands) according to the manufacturer’s instructions and reverse-transcribed using a RevertAid First Strand cDNA Synthesis Kit (Thermo Fisher Scientific). Quantitative polymerase chain reaction (qPCR) reagents were purchased from Thermo Fisher Scientific, and reactions were performed using TaqMan probes and Master Mix (for detection of *HSPA5*, *DDIT3*, *ERN1*) or SYBR Select Master Mix (all other targets) (Applied Biosystems/Thermo Fisher Scientific, Foster City, CA, USA). Alternatively, gene expression was measured using qPCR-based low-density arrays (QIAGEN). The custom array in use was previously designed to cover 84 genes relevant to joint pathology and regulated by proinflammatory stimuli in RA FLS [24, 25].

### Cell viability and apoptosis detection

RA FLS were exposed to thapsigargin at concentrations ranging from 10 nM to 1 μM for 4–24 h. Apoptosis induction was analyzed using the Cell Death Detection ELISA (enzyme-linked immunosorbent assay; Roche Diagnostics/Sigma-Aldrich, Mannheim, Germany) according to the manufacturer’s instructions. Viability was assessed by 3-(4,5-dimethylthiazol-2-yl)-2,5-diphenyltetrazolium bromide (MTT) assay. Following treatment, cells were incubated with 1 mg/ml thiazolyl blue tetrazolium bromide (Sigma-Aldrich) for 1 h at 37 °C. The water-insoluble reaction product was dissolved with isopropanol containing 5 mM HCl and 0.1% Nonidet P-40 and quantified by measuring absorbance at 595 nm.

### ELISA

Cells were stimulated with 10 nM thapsigargin or 1 μg/ml LPS, alone or in combination, for 24 h. Cell-free supernatants were collected, and the concentrations of IL-6 and IL-8 were measured using PeliKine Compact human IL-6 and IL-8 ELISA kits (Sanquin) according to the manufacturer’s instructions.

### Immunoblotting

Cells were stimulated with 10 nM thapsigargin or 1 μg/ml LPS or a combination thereof for 30 minutes and 1, 2, 4, and 8 h, and then they were lysed in modified Laemmli buffer (120 mM Tris-HCl, pH 6.8, 4% SDS, 4% glycerol). Lysates were combined with loading buffer containing β-mercaptoethanol, heat-denatured at 95 °C, resolved by SDS-PAGE electrophoresis, and blotted onto polyvinylidene fluoride membranes. Membranes were blocked with 4% nonfat dry milk for 1 h, followed by overnight probing with primary antibodies recognizing histone 3, IκBα, and phosphorylated forms of JNK, p38 (all from Cell Signaling Technologies, Leiden, The Netherlands), and ERK (Santa Cruz Biotechnology, Dallas, TX, USA). HRP-conjugated secondary antibodies were purchased from Dako/Agilent Technologies (Santa Clara, CA, USA), and proteins were detected using Lumi-Light enhanced chemiluminescence substrate (Roche/Sigma-Aldrich) and the ChemiDoc imaging system (Bio-Rad Laboratories, Hercules, CA, USA). Densitometric analysis of bands was performed with ImageJ software (https://imagej.nih.gov/ij/). Band intensities were normalized to histone 3 signal in the sample and expressed relative to the unstimulated cells.

### Statistical analysis

Data are presented as mean ± SEM. Statistical analysis was performed using Prism 6.02 software (GraphPad Software Inc., La Jolla, CA, USA). The number of replicates in the figure description refers to the number of different FLS donors included in the analysis. For comparison of multiple datasets with a single reference set, repeated measures analysis of variance followed by Dunnett’s post hoc test was used. For comparison between two datasets only, a paired *t* test was used unless otherwise indicated. All tests were two-tailed, and *p* values <0.05 were considered significant.

## Results

### ER stress alone has limited impact on inflammatory gene expression in RA FLS

**Fig. 1:**
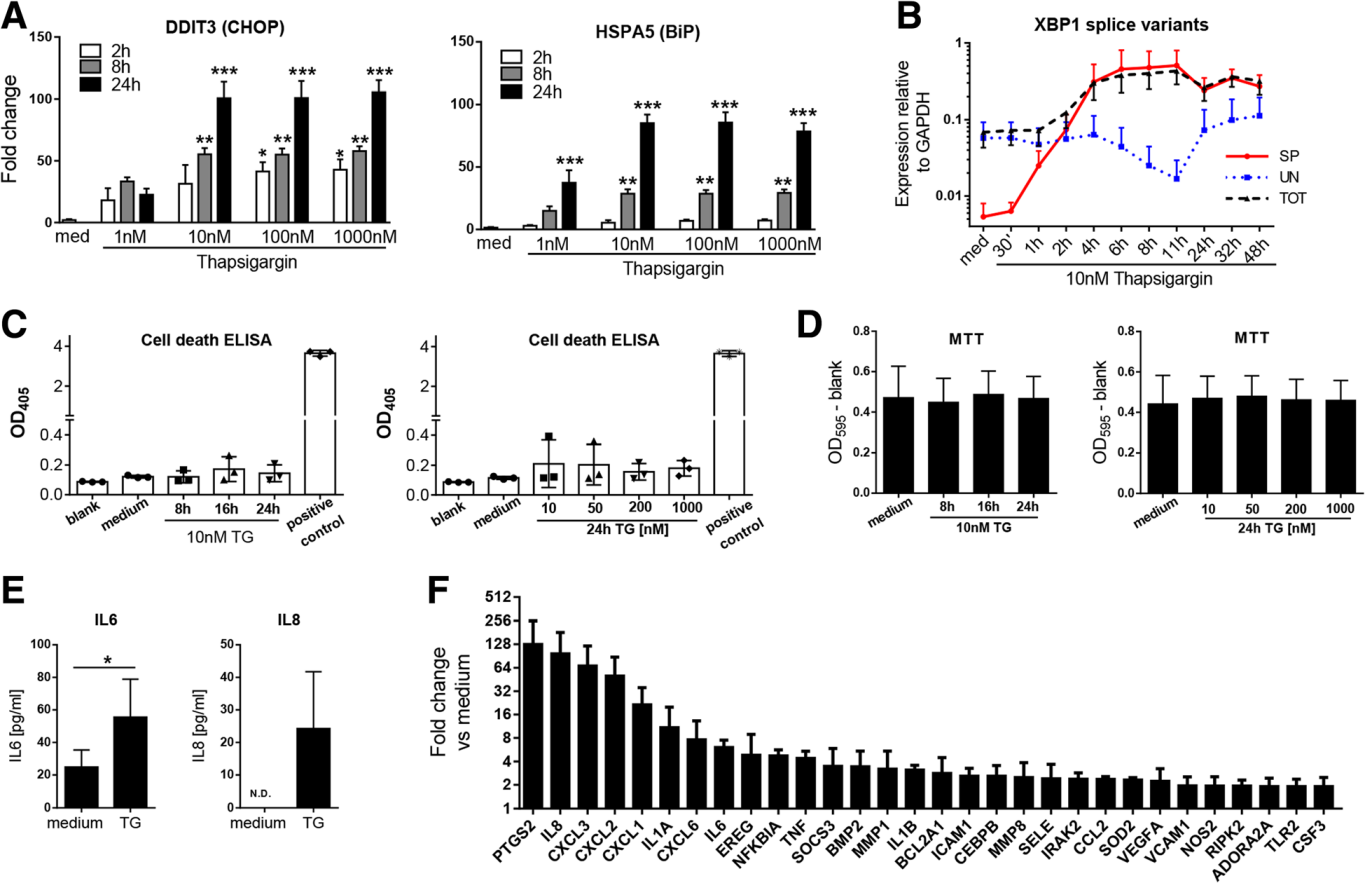
Effects of ER stress on RA FLS inflammatory gene expression and cellular viability. **a**, **b** Expression of ER stress markers (**a**) and XBP1 splice variants (**b**) was measured in three FLS lines after exposure to increasing concentrations of TG (**a**) or constant 10 nM TG (**b**). The significance of changes relative to unstimulated controls was assessed by analysis of variance with post hoc Dunnett’s test. * *p* < 0.05; ** *p* < 0.01; *** *p* < 0.001. **c**, **d** Apoptosis induction and cellular viability were measured using a cell death ELISA (**c**) and an MTT assay (**d**), respectively, after stimulation with 10 nM TG for 8, 16, or 24 h or with increasing concentrations of TG for 24 h. **e** IL-6 and IL-8 in cell culture supernatants (*n* = 3) were measured by ELISA after 24-h incubation with 10 nM TG. **f** FLS (*n* = 3) were incubated with 10 nM TG for 2 h, and expression of 84 inflammatory genes was analyzed by qPCR-based array. Shown are the top 20 genes ranked by mean fold change relative to unstimulated cells. *BiP* Binding immunoglobulin protein, *CHOP* C/EBP homologous protein, *ELISA* Enzyme-linked immunosorbent assay, *ER* Endoplasmic reticulum, *FLS* Fibroblast-like synoviocytes, *GAPDH* Glyceraldehyde 3-phosphate dehydrogenase, *IL* Interleukin, *MTT* 3-(4,5-dimethylthiazol-2-yl)-2,5-diphenyltetrazolium bromide, *N.D.* Not detectable, *OD* Optical density, *qPCR* Quantitative polymerase chain reaction, *RA* Rheumatoid arthritis, *TG* Thapsigargin, *TLR* Toll-like receptor, *TNF* Tumor necrosis factor, *XBP1* X-box binding protein 1

We initiated our studies by analyzing the effects of thapsigargin, a widely used ER stress inducer, on the expression of ER stress markers C/EBP homologous protein (CHOP; *DDIT3*) and BiP (*HSPA5*) in RA FLS. mRNA levels of these genes were significantly increased in a dose- and time-dependent manner following thapsigargin treatment (Fig. 1a). Induction was readily observed 2 h after treatment and continued to increase for as long as 24 h. Notably, although thapsigargin is typically used at micromolar concentrations [17, 19], 10 nM thapsigargin was sufficient for maximum induction of *DDIT3* and *HSPA5* in FLS*.* To quantitatively assess signaling emanating directly from the ER, we developed an assay in which expression of *XBP1* splice variants is followed by real-time PCR using primers recognizing only its IRE1α-processed (spliced) or unprocessed (unspliced) versions or detecting both variants indiscriminately (total). At baseline, most of *XBP1* was in its unspliced form, confirming a low level of IRE1α activity (Fig. 1b). The amount of spliced *XBP1* increased exponentially 30 minutes after stimulation with 10 nM thapsigargin and soon approximated the amount of total *XBP1*, indicating sustained and maximal UPR signaling. Consistent with previous studies [17–19], we observed no apoptotic effect of ER stress in RA FLS for up to 24 h after treatment with thapsigargin over a range of 10 nM to 1 μM (Fig. 1c). Similarly, thapsigargin had no effect on RA FLS cellular viability, as measured by MTT assay (Fig. 1d). Thus, RA FLS are biochemically and transcriptionally sensitive to ER stress in the absence of effects on cellular survival.

We next examined if ER stress leads to modulation of inflammatory gene expression in RA FLS. In initial experiments, we observed increased amounts of IL-6 and IL-8 protein present in the media of cells exposed to thapsigargin (Fig. 1e). Although statistically significant, the concentrations were low compared with cytokine production induced by agonists such as IL-1β, LPS, and TNF (*see below* and data not shown). We therefore analyzed the expression of 84 genes responsive to proinflammatory stimuli in FLS. Surprisingly, only a few genes, such as *PTGS2*, *IL8*, and a subset of chemokine (C-X-C motif) ligand (CXCL) chemokines, appeared to be substantially regulated by thapsigargin treatment (Fig. 1f). We subsequently validated these results using tunicamycin, a molecule that causes ER stress by an unrelated mechanism, and obtained similarly modest changes in the gene expression pattern, despite clear induction of UPR signaling (Additional file 1).

### ER stress cooperates with TLR signaling to regulate inflammatory gene expression

**Fig. 2:**
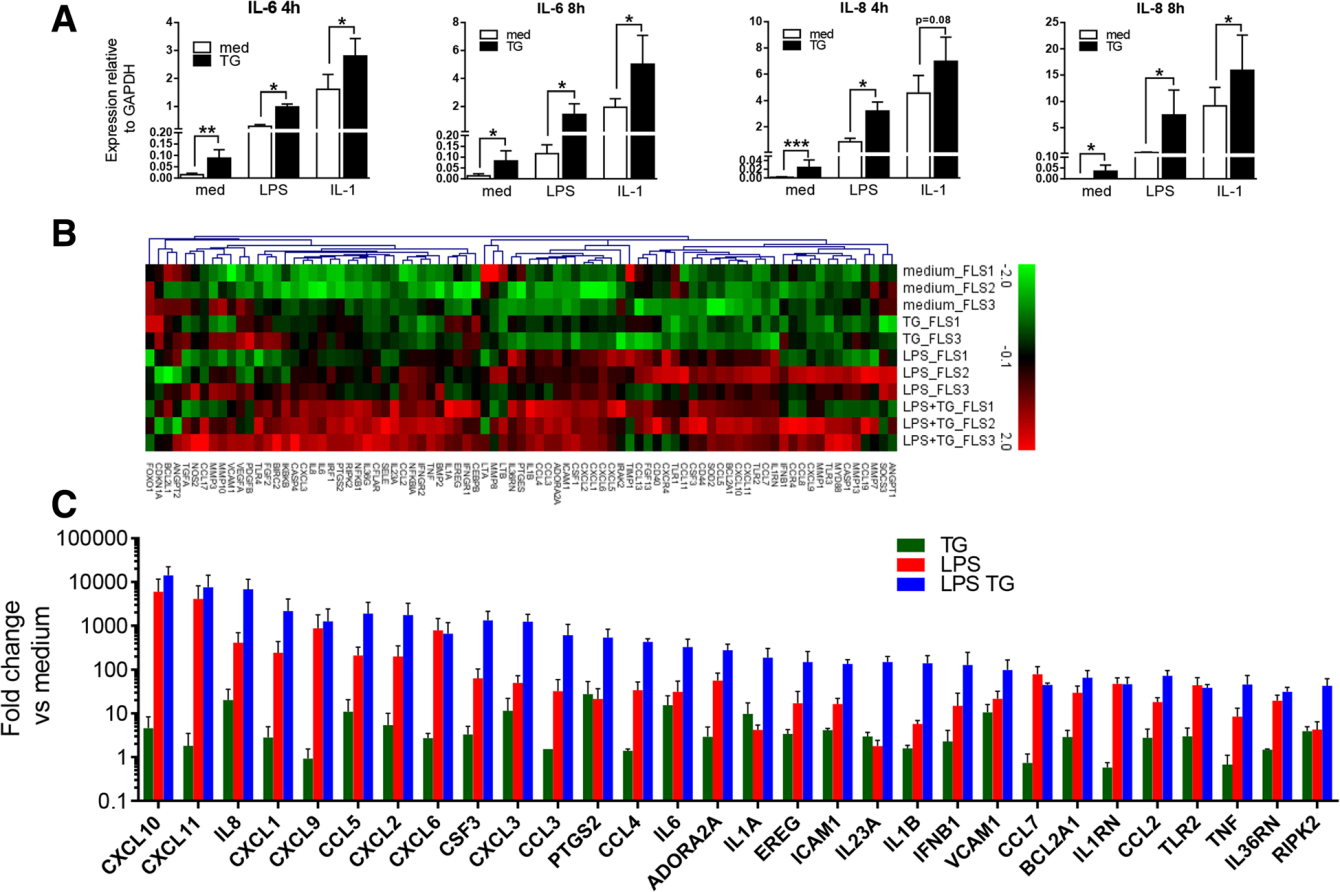
Synergism between ER stress and other stimuli. **a** FLS (*n* = 4) were left untreated or stimulated with 1 μg/ml LPS or 1 ng/ml IL-1β in the presence or absence of 10 nM TG. Expression of *IL6* and *IL8* was measured by qPCR 4 h or 8 h after stimulation. **b**, **c** FLS (*n* = 3, *n* = 2 for TG alone) were stimulated with 10 nM TG or 1 μg/ml LPS in the presence or absence of 10 nM TG for 8 h. Expression of 84 inflammatory genes was monitored by qPCR-based array. Heat map (**b**) depicts per-gene z-scores of log-scaled expression values relative to GAPDH, and bar graph (**c**) presents fold changes relative to unstimulated cells for 30 genes with the highest overall level of regulation (mean across all conditions). *ER* Endoplasmic reticulum, *FLS* Fibroblast-like synoviocytes, *GAPDH* Glyceraldehyde 3-phosphate dehydrogenase, *IL* Interleukin, *LPS* Lipopolysaccharide, *qPCR* Quantitative polymerase chain reaction, *TG* Thapsigargin

**Fig. 3:**
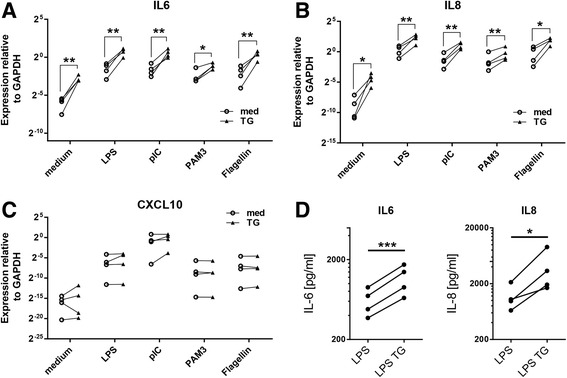
Synergism between ER stress and multiple TLR ligands. mRNA expression of *IL6* (**a**), *IL8* (**b**), and *CXCL10* (**c**) in RA FLS was measured by qPCR after 4-h stimulation with the indicated TLR ligands in the presence or absence of 10 nM TG. **d** IL-6 and IL-8 protein production in response to combined treatment with LPS and TG was measured by ELISA. * *p* < 0.05; ** *p* < 0.01; *** *p* < 0.001. *CXCL* Chemokine (C-X-C motif) ligand, *ELISA* Enzyme-linked immunosorbent assay, *ER* Endoplasmic reticulum, *FLS* Fibroblast-like synoviocytes, *GAPDH* Glyceraldehyde 3-phosphate dehydrogenase, *IL* Interleukin, *LPS* Lipopolysaccharide, *mRNA* Messenger RNA, *pIC* Polyinosinic:polycytidylic acid, *qPCR* Quantitative polymerase chain reaction, *RA* Rheumatoid arthritis, *TG* Thapsigargin, *TLR* Toll-like receptor

We next examined the capacity of ER stress to interact with other inflammatory stimuli by incubating RA FLS with IL-1β or LPS with or without thapsigargin. Thapsigargin alone again had significant, although minor, effects on *IL6* (Fig. 2a, *left panels*) and *IL8* (Fig. 2a, *right panels*) expression. However, we observed highly consistent trends toward higher expression of both analytes when LPS or IL-1β was used in combination with ER stress induction, with the differences particularly apparent in the context of LPS stimulation. Expanding our analyses to a larger set of genes, we found that ER stress enhanced the expression of most of the transcripts regulated by LPS (Fig. 2b). We observed that 53% of genes showed increased induction of greater than twofold relative to LPS alone, and over one-third (34.9%) of them showed greater than fivefold increases (Fig. 2c; note logarithmic scale). Finally, we examined whether synergistic regulation of gene expression by ER stress could also be observed for other TLR ligands (Fig. 3). Of genes for which strong synergism could be observed between ER stress and LPS, such as *IL6* (Fig. 3a), *IL8* (Fig. 3b), *CCL3*, *PTGS2*, *TNF*, and *IFNB* (data not shown), we observed a similar synergistic effect between ER stress and other TLR ligands, including pI:C, Pam3CSK4, and flagellin. Consistently, no combinatorial effect between TLR ligands and thapsigargin was observed for *CXCL10* (Fig. 3c) and other genes (data not shown) that were refractory to modulation by ER stress during LPS stimulation. Changes observed in LPS-induced gene transcription in the presence of ER stress were functionally relevant because we could detect significantly elevated levels of IL-6 and IL-8 in cell culture supernatants of RA FLS when cells were exposed to both LPS and thapsigargin (Fig. 3d).

### Effects of ER stress on inflammatory gene expression in RA FLS depend primarily on changes in mRNA stability

We hypothesized that ER stress, while having little effect on its own, may act by increasing the magnitude or the duration of signaling initiated by other events. Using TLR4 stimulation as a model, we analyzed activation of NF-κB and MAP kinase pathways (Additional file 2: Figure S2a), but we failed to observe pronounced modulatory effects of thapsigargin, although quantitative analysis of multiple experiments demonstrated that p38 activation and degradation of IκBα were maintained longer during ER stress (Additional file 2: Figure S2b). Thus, although ER stress may lead to a slight prolongation of activation of inflammatory signaling pathways, the magnitude of these changes is insufficient to explain profound modulation of inflammatory gene expression.

**Fig. 4:**
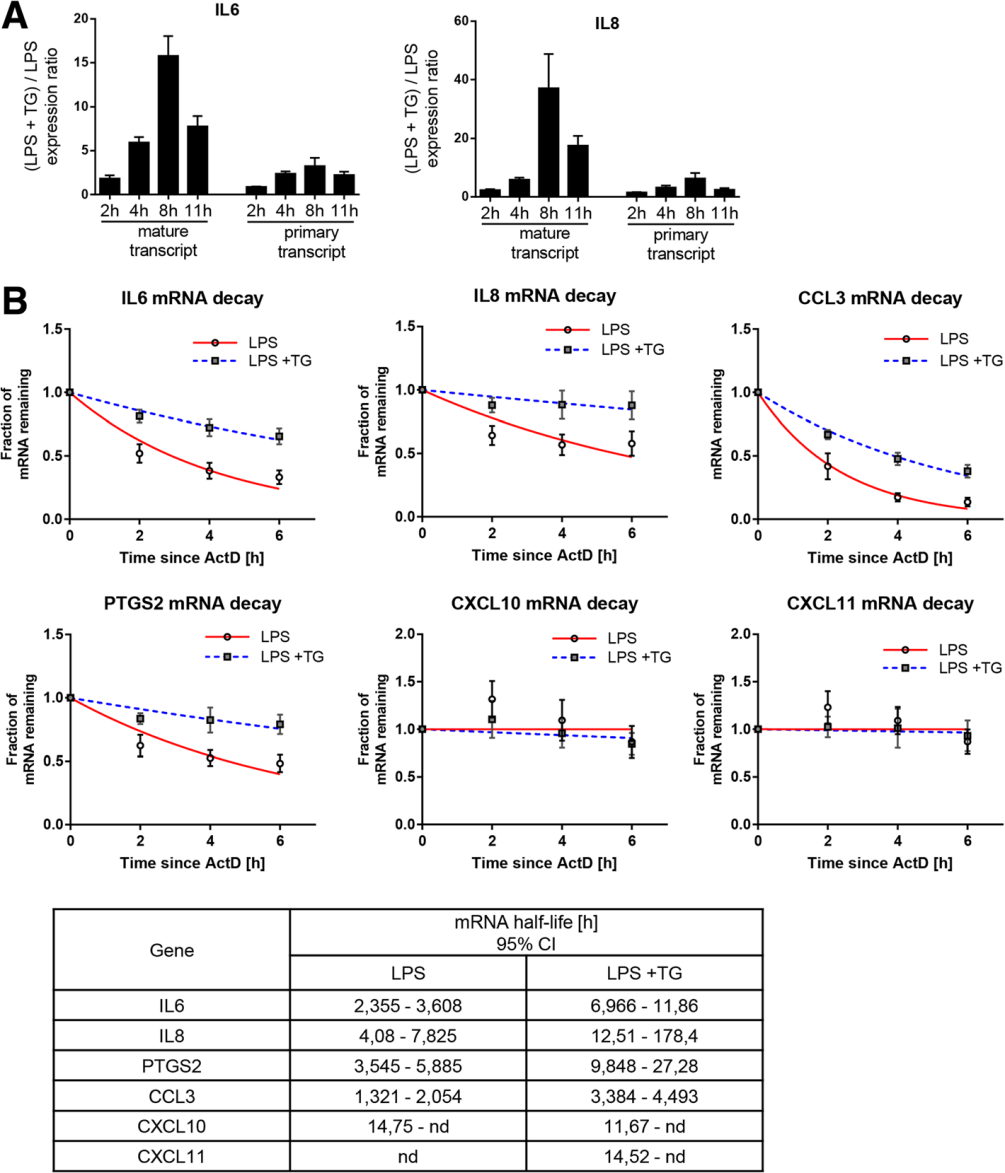
Effects of ER stress on gene transcription and mRNA stability in RA FLS. **a** FLS (*n* = 3) were stimulated with 1 μg/ml LPS in the presence or absence of 10 nM TG for the indicated amount of time. mRNA expression of mature and primary forms of transcripts of *IL6* and *IL8* was measured by qPCR. Shown is the ratio between expression observed in both experimental conditions at each time point. **b** FLS (*n* = 8) were stimulated with 1 μg/ml LPS in the presence or absence of 10 nM TG for 4 h, followed by incubation with 10 μg/ml ActD to induce transcriptional block. Cells were lysed at the indicated time points after addition of ActD, and the amount of transcript for each gene remaining in cells was analyzed by qPCR. Data are presented as a fraction of transcript detectable at each time point relative to the moment immediately after addition of ActD. Table shows 95% CIs of transcript half-life calculated using Prism software (GraphPad Software). *ActD* Actinomycin D; *CXCL* Chemokine (C-X-C motif) ligand, *ER* Endoplasmic reticulum, *FLS* Fibroblast-like synoviocytes, *IL* Interleukin, *LPS* Lipopolysaccharide, *mRNA* Messenger RNA, *qPCR* Quantitative polymerase chain reaction, *RA* Rheumatoid arthritis, *TG* Thapsigargin

Next, we examined whether differences in gene expression could be explained by differences in transcriptional activity at their loci. We used the amount of primary transcripts, nascent transcripts produced by RNA polymerase before intronic sequence excision, as a surrogate measure of the transcription rate and compared the kinetics of expression of the primary transcripts and mature mRNAs encoding *IL6* and *IL8*. After 8 h of stimulation, expression of mature forms of *IL6* and *IL8* was approximately 15 and 40 times higher, respectively, in cells treated with the combination of LPS and thapsigargin than with LPS alone (Fig. 4a). The corresponding values for the primary transcript were much lower indicating that an increase in the transcription rate can account for only a fraction of the elevated mRNA expression during combined LPS and thapsigargin treatment. Because these results pointed to the importance of posttranscriptional regulatory mechanisms, we investigated possible differences in mRNA decay rates. Cells were stimulated with LPS or LPS and thapsigargin for 4 h, at which point further transcription was blocked and the amount of mature mRNA remaining in the cells was followed over time. We observed that the rates of mRNA decay of genes synergistically regulated by LPS and ER stress, including *IL6*, *IL8*, *CCL3*, and *PTGS2*, were significantly slowed by ER stress as compared with rates observed with LPS alone (Fig. 4b and c). In contrast, ER stress had little to no effect on the stability of mRNA encoding genes refractory to modulation by thapsigargin, such as *CXCL10* or *CXCL11* (Fig. 4b and c). By using tunicamycin as an alternative ER stress inducer, we validated that both the observed synergy and stabilization of cytokine mRNAs are caused by the ER stress itself and not by compound-specific effects of thapsigargin (Additional file 3: Figure S3).

Because regulation of mRNA decay in response to inflammatory stimuli is known to be strongly influenced by MAP kinase signaling tone [26], we decided to reexamine MAP kinases’ potential involvement. SB202190, a specific p38 inhibitor, significantly accelerated mRNA decay in cells exposed to both LPS alone and LPS in combination with thapsigargin (Additional file 4: Figure S4a). It was, however, insufficient to equalize the observed decay rates, suggesting that the contribution of ER stress to mRNA stabilization does not rely on changes in p38 activity. Inhibition of the JNK and ERK pathways had similarly little effect on narrowing the differences in the fraction of mRNA detectable in cells after 2 h of actinomycin D chase (Additional file 4: Figure S4b).

### Enhanced mRNA stability of proinflammatory genes during ER stress is observed in stromal but not myeloid cells

**Fig. 5:**
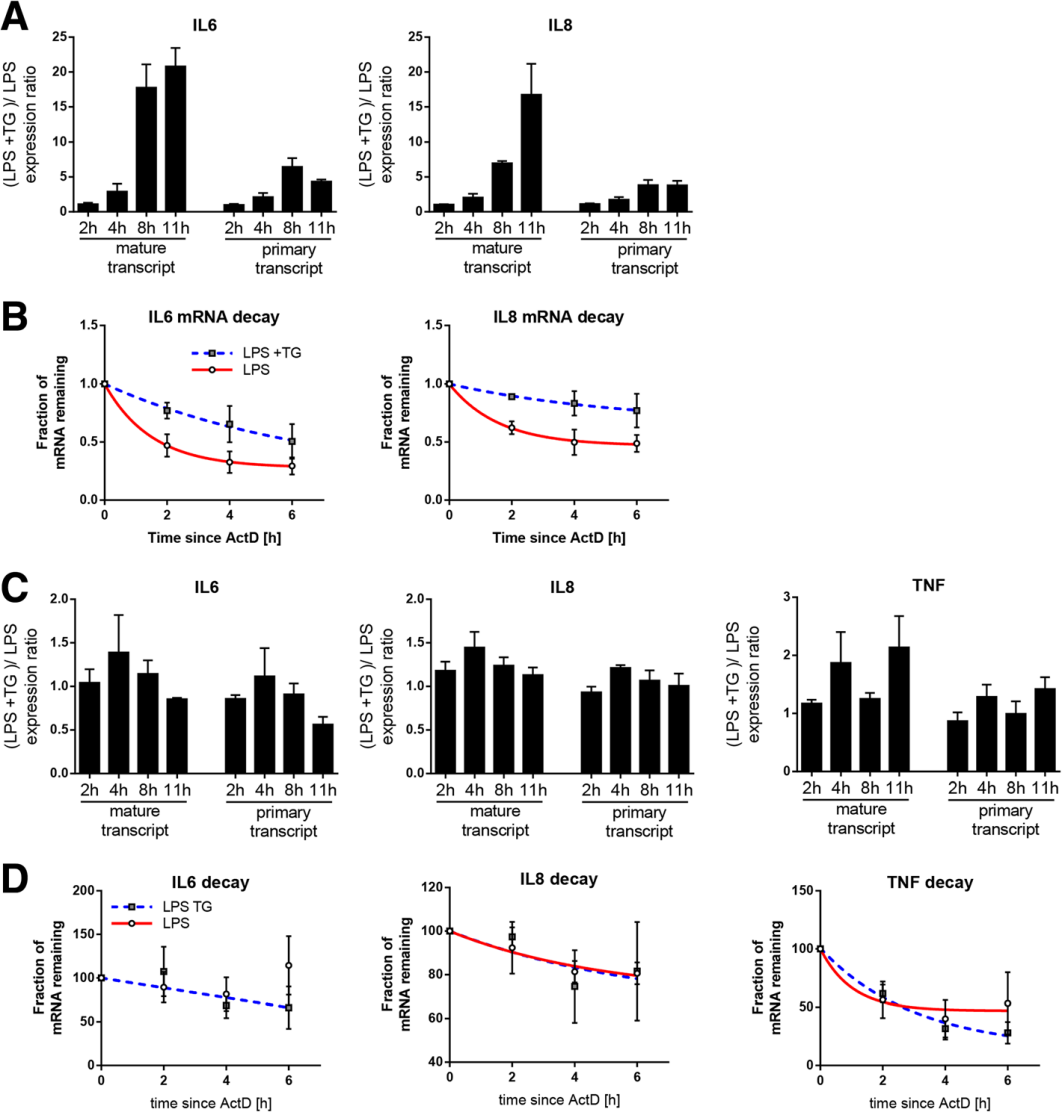
Synergism between ER stress and LPS in dermal fibroblasts and macrophages. **a**, **c** Human dermal fibroblasts (**a**, *n* = 3) or GM-CSF-differentiated macrophages (**c**, *n* = 3) were stimulated with 1 μg/ml LPS in the presence or absence of 10 nM TG for the indicated amount of time. mRNA expression of mature and primary forms of transcripts of *IL6*, *IL8*, and *TNF* was measured by qPCR. Shown is the ratio between expression observed in both experimental conditions at each time point. **b**, **d** Human dermal fibroblasts (**b**, *n* = 3) or GM-CSF-differentiated macrophages (**d**, *n* = 3) were stimulated with 1 μg/ml LPS in the presence or absence of 10 nM TG for 4 h, followed by incubation with 10 μg/ml ActD to induce transcriptional block. Cells were lysed at the indicated time points after addition of ActD, and the amount of transcript for each gene remaining in cells was analyzed by qPCR. Data are presented as a fraction of transcript detectable at each time point relative to the moment immediately after addition of ActD. *ActD* Actinomycin D, *ER* Endoplasmic reticulum, *GM-CSF* Granulocyte-macrophage colony-stimulating factor, *IL* Interleukin, *LPS* Lipopolysaccharide, *mRNA* Messenger RNA, *qPCR* Quantitative polymerase chain reaction, *TG* Thapsigargin, *TNF* Tumor necrosis factor

To establish whether the ability of ER stress to regulate inflammatory gene expression was limited to FLS, we analyzed the effects of LPS treatment combined with ER stress in DF, representing another fibroblastic cell, and macrophages, representing both an unrelated lineage and a major cellular constituent of the inflamed synovial membrane. In DF, coincubation of cells with thapsigargin and LPS resulted in enhanced expression of *IL6* and *IL8* similar to that observed in FLS (Fig. 5a). Also, although the kinetics of primary transcript expression failed to explain the level of synergy in the mature transcript (Fig. 5a), we again observed increased mRNA stability of these cytokines (Fig. 5b). The presence of thapsigargin did not result in similarly high changes in the expression of *IL6*, *IL8*, and *TNF* in macrophages (Fig. 5c). The transcription rate was only marginally affected, and no discordance between primary and mature transcript kinetics was observed. In line with these results, we failed to observe stabilization of mRNA in macrophages treated with LPS and thapsigargin (Fig. 5d). Also, at higher concentrations of thapsigargin, we did not notice synergistic effects with LPS, but we observed strong cytotoxic effects in macrophages (data not shown). Our results suggest that in RA synovial tissue, ER stress contributes to local inflammation primarily through its effects on FLS, rather than myeloid cells, by promoting mRNA stabilization of genes relevant to pathology.

## Discussion

ER stress plays an important role in both physiological and pathological immune responses, and RA and OA synovia are distinguished by a strong UPR signature [14]. Similarly, macrophages isolated from RA patient synovial fluid were demonstrated by several groups to bear signs of ER stress, as compared with both peripheral blood monocyte-derived macrophages or macrophages isolated from OA patient synovial fluid [14, 16]. In the present study, we demonstrate that also stromal cells, FLS, are readily responsive to ER stress induction, but by itself this has little significant effect on cellular activation, nor does it affect viability. Instead, ER stress primes stromal cells for enhanced cytokine and chemokine production in the presence of other agonistic signals. The magnitude and range of this synergistic response are in stark contrast with the negligible changes in cytokine expression induced by thapsigargin alone, and they are a result of effects on transcriptional rates, to some extent, and primarily on mRNA decay rates of inflammatory genes.

In regard to cell survival, RA FLS were previously described as more resistant to such challenge than OA FLS, with altered expression of CHOP and synoviolin postulated as possible mechanisms [17, 18]. Similarly, the ER chaperone BiP has been identified as an important survival factor for stressed synoviocytes, and its expression regulates joint destruction in animal models [14]. On the other hand, inflammatory responses to ER stress in FLS have been scarcely studied so far. Contrary to freshly isolated synovial fluid macrophages, cultured RA FLS do not show signs of increased ER stress [20], although they upregulate UPR-related genes more readily than OA FLS in response to a variety of stimuli [14]. Analogously to similar observations in macrophages [9], a possible effect of TLR-dependent XBP1 activation on gene expression has been proposed in RA FLS [20]. However, following stimulation with LPS alone, we have observed only minor differences in the amount and fraction of *XBP1* existing in the spliced form, indicating no significant changes in UPR signaling (data not shown).

Our data suggest that enhanced mRNA stability is a major contributor to the increased level of gene expression during ER stress. A growing number of reports underscore the importance of mRNA stability regulation during chronic synovitis in RA. In particular, Loupasakis et al. [27] recently demonstrated mRNA stabilization as a crucial factor shaping the FLS transcriptome during long-term exposure to TNF, with a strong influence on *IL6*, *IL8*, *CCL2*, *PTGS2*, and other genes with pathogenic potential.

Intriguingly, ER stress has long been known to impact the mRNA stability of certain genes via regulated IRE1α-dependent degradation [28]. In such cases, activated IRE1α was shown to splice not only *XBP1* but also several other mRNAs, resulting in their accelerated decay. However, the idea that ER stress might conversely contribute to inflammation by stabilizing cytokine mRNA has not previously been explored. Regulation of cytokine expression through changes in mRNA stability depends primarily on the presence of adenylate- and uridylate-rich elements in their sequences. These are recognized by adenylate- and uridylate-rich element-binding proteins (ABPs) whose expression and activity are tightly regulated and can lead to both positive and negative regulation of mRNA half-life [29]. We have screened possible candidate ABPs, including BRF1 (*ZFP36L1*), BRF2 (*ZFP36L2*), AUF1 (*HNRNPD*), TTP (*ZFP36*), HuR (*ELAVL1*) and *KHSRP*, using small interfering RNA-mediated knockdown (data not shown), but we were unsuccessful in mimicking or significantly modulating the effects of combined LPS and thapsigargin stimulation by their independent targeting. Additionally, inhibition of conventional pathways involved in ABP-mediated decay [26], such as p38, ERK, and JNK, did not block a positive effect of ER stress on mRNA stability. These observations indicate that additional regulatory layers, such as microRNAs or components of nonsense-mediated decay, may be implicated.

The observation that ER stress regulates mRNA stability in DF is similarly novel, suggesting a shared mode of stromal cell response to suspected injury by preparing to mount a rapid inflammatory response if further danger signals appear in the environment. This may be relevant to rheumatic diseases other than RA characterized by skin involvement, such as psoriatic arthritis and systemic sclerosis. In this regard, the role of TLR ligands and ER stress in systemic sclerosis has been described extensively (reviewed in [30, 31]), and it will be of interest to determine whether ER stress-dependent regulation of gene expression contributes to the acquisition of the profibrotic phenotype in these patients.

Our inability to observe a similar effect of ER stress on mRNA stability in macrophages was surprising, given the available literature. For example, the IRE1α-XBP1 signaling pathway was shown to be a critical element of macrophage responses to TLR ligation [9], and myeloid-specific knockout of IRE1α ameliorated disease severity in the K/BxN serum-induced arthritis model [16]. Although the primary focus of these previous studies was the role of IRE1α during TLR stimulation alone, an enhancement of LPS-induced cytokine expression during ER stress in murine bone marrow-derived macrophages was noted, and a similar finding was observed in human macrophages [9]. The discrepancy in macrophage responses to ER stress between these studies and ours, where we also noted a sensitivity of macrophages to LPS and ER stress-induced apoptosis, may be a result of differences in tissue- and polarization-specific macrophage responses. In line with this, it was previously observed that resident and thioglycolate-elicited peritoneal macrophages show opposite patterns of regulation of *CXCL1* during stimulation with LPS and thapsigargin [32]. Our results suggest that in RA synovial tissue, the IRE1α-XBP1 axis might contribute to macrophage responses to TLR signaling in the absence of induction of ER stress, whereas in stromal cells, TLR stimulation in the presence of ER stress amplifies cytokine and chemokine production.

## Conclusions

Whereas a strong ER stress signature is a distinguishing feature of RA synovium, the understanding of its capacity to influence pathological processes was incomplete. Specifically in the case of stromal cells, the effects of ER stress reported in the literature that could be relevant in a disease setting are linked to the RA FLS intrinsic resistance to apoptosis and were not known to contain an inflammatory component. In our present study, however, we identify a novel regulatory mechanism relying on interactions between stromal cells, ER stress, and molecular danger signals with potential to profoundly affect the course of the locally developed inflammation. We propose a model in which cytokine transcription is initiated by an external trigger rather than by ER stress itself, with the latter being instead responsible for promoting mRNA stability. Combination of both inputs leads to significant augmentation of the overall response in stressed cells. Further characterization of this mechanism may lead to identification of molecular targets relevant for a range of immune-mediated inflammatory diseases characterized by synovial and connective tissue involvement.

## Additional files

Additional file 1: Figure S1. Tunicamycin treatment results in ER stress induction with gene expression pattern similar to that caused by thapsigargin. (TIF 295 kb)

Additional file 2: Figure S2. ER stress effects on TLR-proximal signaling pathways. (TIF 875 kb)

Additional file 3: Figure S3. Tunicamycin-induced ER stress synergizes with LPS in regulation of gene expression and restricts mRNA decay in the same way as when induced by thapsigargin. (TIF 391 kb)

Additional file 4: Figure S4. Modulation of mRNA decay by ER stress is MAPK-independent. (TIF 571 kb)

## Abbreviations

ABP: Adenylate- and uridylate-rich element-binding protein
ActD: Actinomycin D
BiP: Binding immunoglobulin protein
CHOP: C/EBP homologous protein
CXCL: Chemokine (C-X-C motif) ligand
DF: Dermal fibroblasts
ELISA: Enzyme-linked immunosorbent assay
ER: Endoplasmic reticulum
ERK: Extracellular signal-regulated kinase
FLS: Fibroblast-like synoviocytes
GAPDH: Glyceraldehyde 3-phosphate dehydrogenase
GM-CSF: Granulocyte-macrophage colony-stimulating factor
IκBα: Nuclear factor of kappa light polypeptide gene enhancer in B-cells inhibitor, alpha
IL: Interleukin
IMDM: Iscove’s modified Dulbecco’s medium
IRE1α: Inositol-requiring enzyme 1α
JNK: c-Jun N-terminal kinase
LPS: Lipopolysaccharide
MAP: Mitogen-activated protein
mRNA: Messenger RNA
MTT: 3-(4,5-dimethylthiazol-2-yl)-2,5-diphenyltetrazolium bromide
NF-κB: Nuclear factor-κB
OA: Osteoarthritis
OD: Optical density
PERK: Protein kinase R-like endoplasmic reticulum kinase
pI:C: Polyinosinic:polycytidylic acid
qPCR: Quantitative polymerase chain reaction
RA: Rheumatoid arthritis
TG: Thapsigargin
TLR: Toll-like receptor
TNF: Tumor necrosis factor
UPR: Unfolded protein response
XBP1: X-box binding protein 1
