# Supplementary figures and images for: Endoplasmic reticulum stress cooperates with Toll-like receptor ligation in driving activation of rheumatoid arthritis fibroblast-like synoviocytes
Source: Arthritis Res Ther. 2017 Sep 18;19:207. doi: 10.1186/s13075-017-1386-x (PMC5604427; doi:10.1186/s13075-017-1386-x)

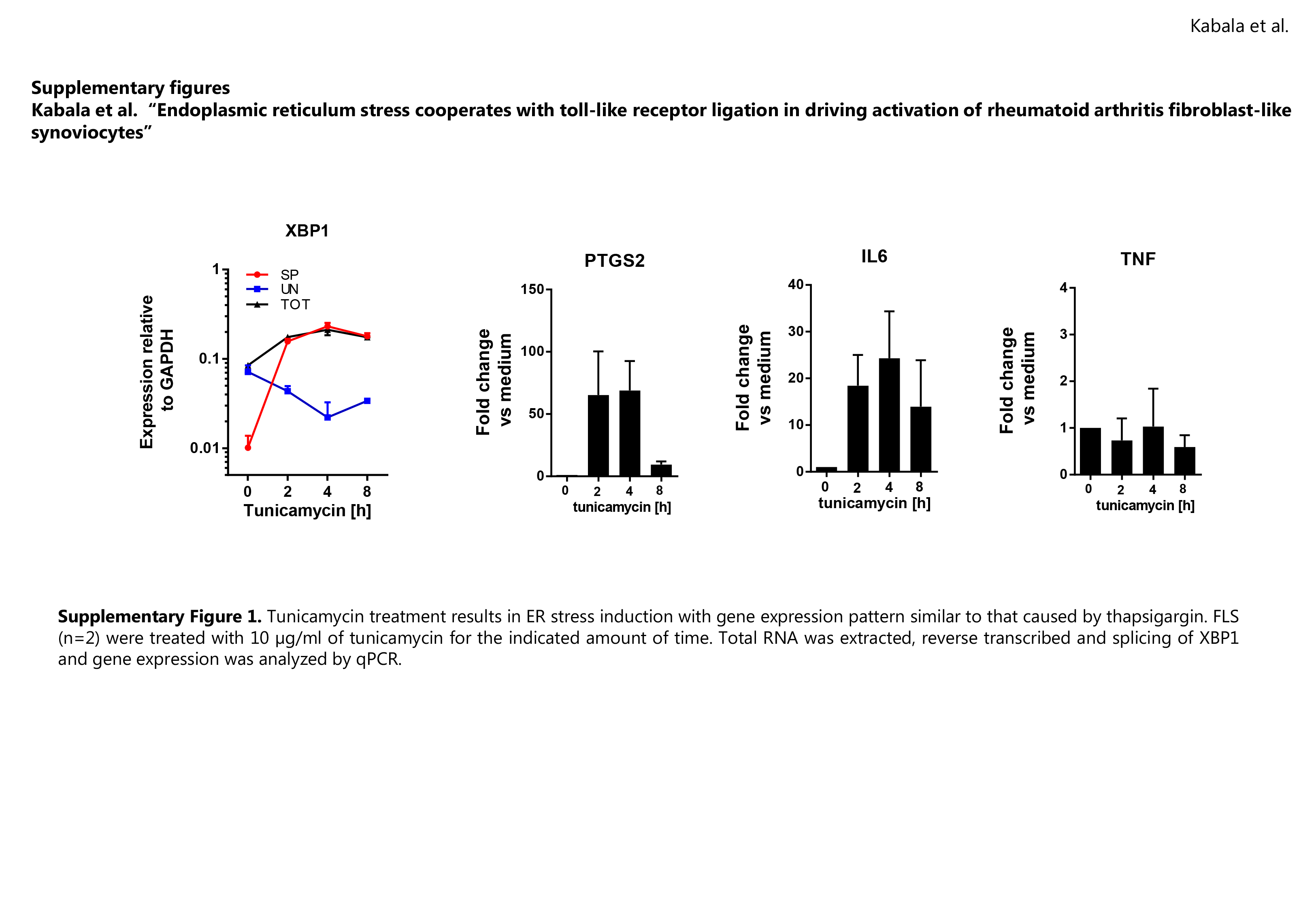

Supplement: Supplementary file 1 — Tunicamycin treatment results in ER stress induction with gene expression pattern similar to that caused by thapsigargin. (TIF 295 kb) [file 13075_2017_1386_MOESM1_ESM.tif]

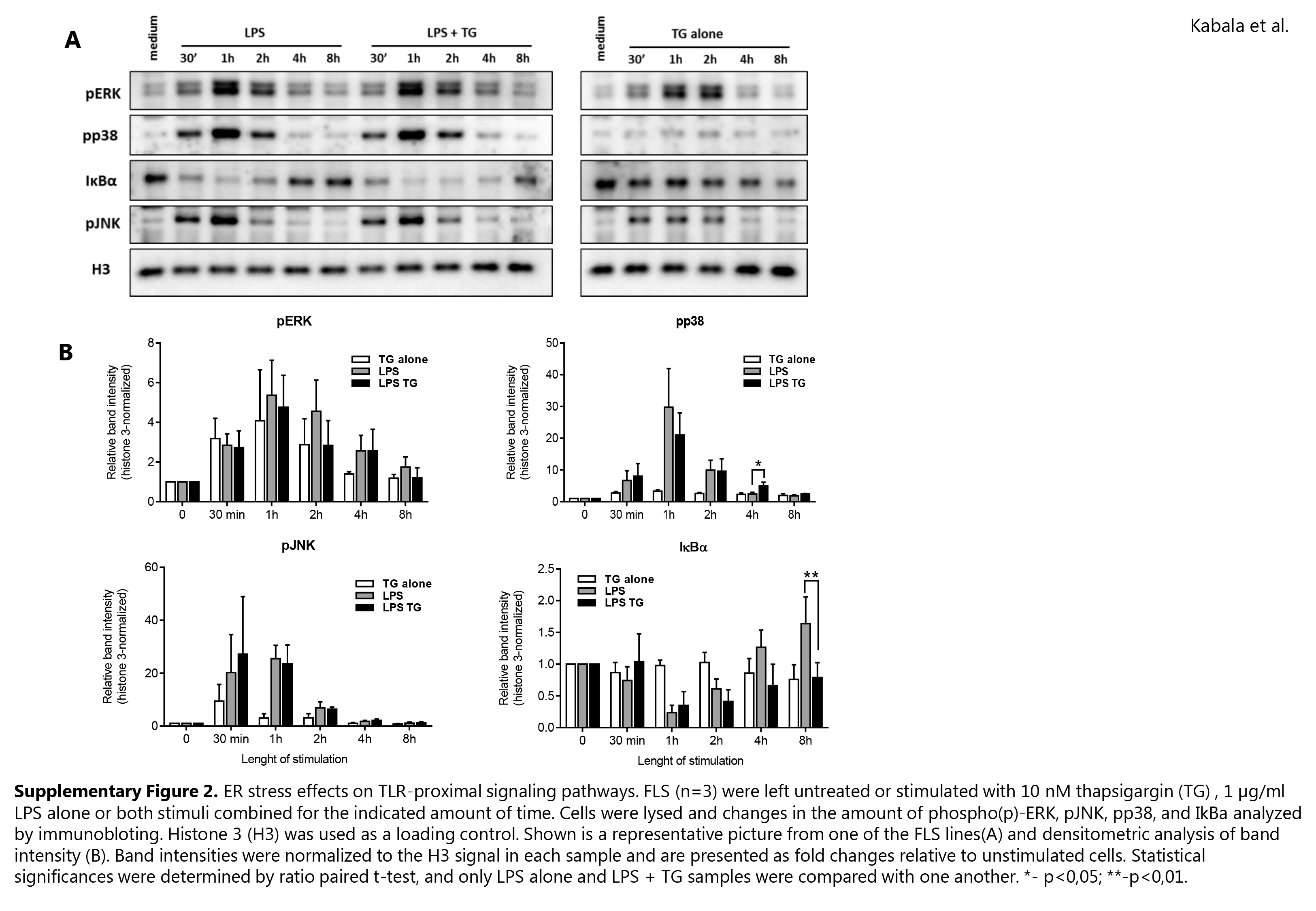

Supplement: Supplementary file 2 — ER stress effects on TLR-proximal signaling pathways. (TIF 875 kb) [file 13075_2017_1386_MOESM2_ESM.tif]

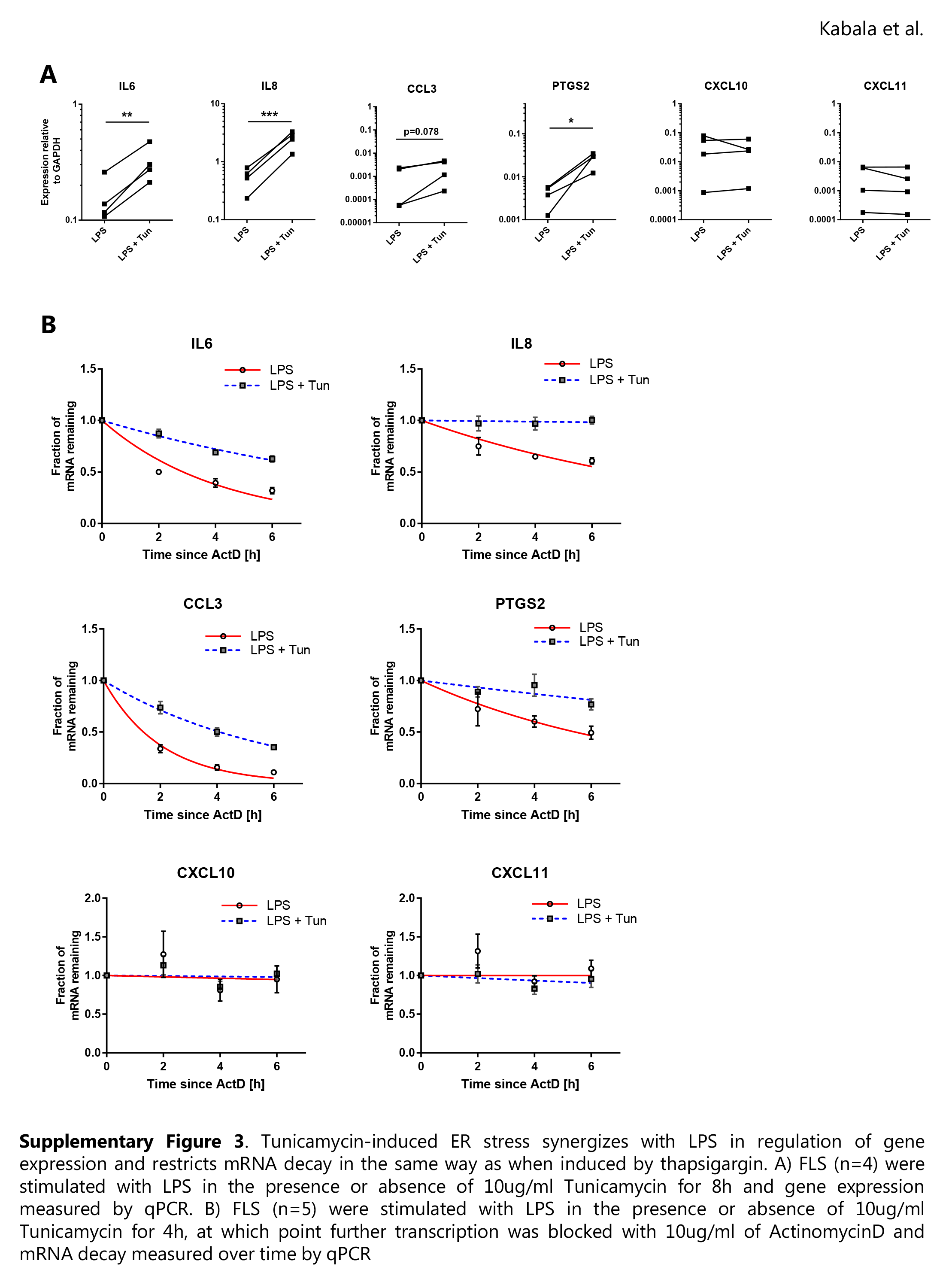

Supplement: Supplementary file 3 — Tunicamycin-induced ER stress synergizes with LPS in regulation of gene expression and restricts mRNA decay in the same way as when induced by thapsigargin. (TIF 391 kb) [file 13075_2017_1386_MOESM3_ESM.tif]

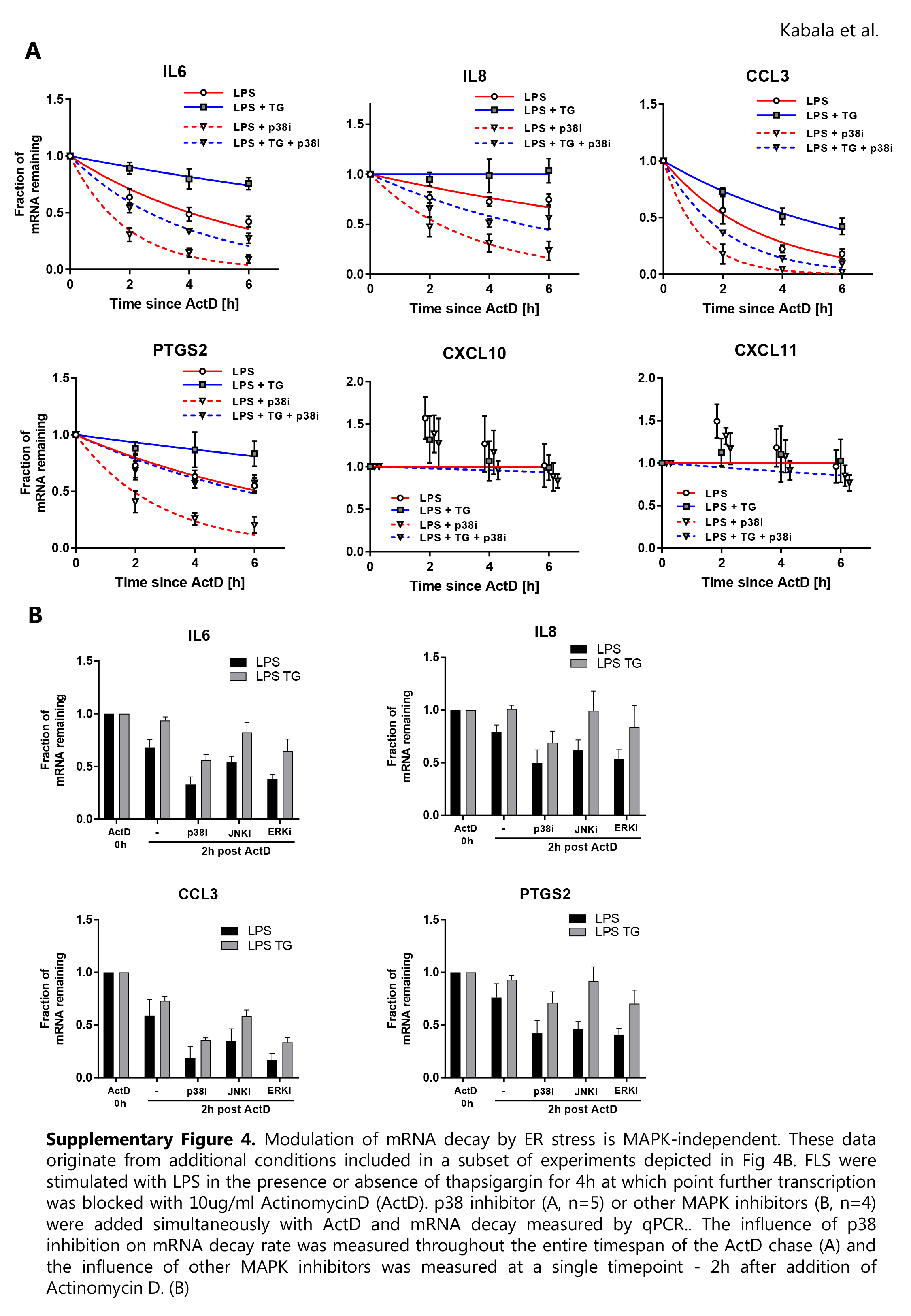

Supplement: Supplementary file 4 — Modulation of mRNA decay by ER stress is MAPK-independent. (TIF 571 kb) [file 13075_2017_1386_MOESM4_ESM.tif]
